# Rapid Bactericidal Activity of *Punica granatum* L. Peel Extract: A Natural Alternative for Mastitis Prevention in Dairy Cattle

**DOI:** 10.3390/molecules30112387

**Published:** 2025-05-29

**Authors:** Carenn Rodrigues e Almeida Silva, Camila Silva Vidal, Sergio Martins de Andrade Filho, Izabela Martins Agatão, Lidiane Coelho Berbert, João Bosco de Salles, Alexander Machado Cardoso, Ricardo Machado Kuster, Cristiane Pimentel Victório, Maria Cristina de Assis

**Affiliations:** 1 Department of Biology, Rio de Janeiro State University, Rio de Janeiro 23070-200, Brazil; carennrasbiopesq@gmail.com (C.R.e.A.S.); camila.s.vidal2@gmail.com (C.S.V.); sergioandrade.ld@gmail.com (S.M.d.A.F.); martins.izabela123@gmail.com (I.M.A.); joao.salles@uerj.br (J.B.d.S.); cristiane.victorio@uerj.br (C.P.V.); assis.maria@uerj.br (M.C.d.A.); 2 Department of Chemistry, Federal University of Espírito Santo (UFES), Vitória 29047-105, Brazil; ricardo.m.kuster@ufes.br

**Keywords:** bovine mastitis, disinfectants, plant-based antimicrobial, pre- and post-dipping

## Abstract

The increasing prevalence of bacterial resistance to conventional disinfectants and antibiotics has intensified the search for effective, natural alternatives in the dairy industry. This study evaluates the bactericidal efficacy of *Punica granatum* L. (pomegranate) peel ethanolic extract, focusing on its application in pre- and post-dipping procedures for mastitis prevention. The extract exhibited potent activity against *Escherichia coli* and *Staphylococcus aureus*, two major mastitis pathogens. At a concentration of 10 mg/mL, the extract induced significant membrane disruption within 30 s of exposure, as evidenced by propidium iodide uptake and elevated extracellular DNA levels (*Escherichia coli*: 64.25 ng/μL; *Staphylococcus aureus*: 83.25 ng/μL) compared to controls (11.20 ng/μL and 35.20 ng/μL, respectively; *p* < 0.05). Complete growth inhibition (100%) was achieved within 30 s at 25 and 50 mg/mL, matching the efficacy of commercial chlorhexidine and high-concentration hypochlorite. Phytochemical analysis identified punicalagin as the predominant bioactive compound. These findings establish *Punica granatum* peel extract as a fast-acting bactericidal agent, exhibiting an efficacy comparable to or exceeding that of conventional disinfectants. Its rapid action and plant-based origin highlight its potential as a viable alternative for the prevention and control of bovine mastitis in dairy farming.

## 1. Introduction

Bovine mastitis is one of the most prevalent and economically significant diseases in cattle farming. It is characterized by inflammation of the udder, primarily caused by bacterial infections, leading to compromised udder health, reduced milk yield, and diminished milk quality [1]. These infections can enter the udder through the teat canal during milking or from environmental sources like contaminated bedding or equipment. Due to its recurrent nature, mastitis remains a persistent challenge for the dairy industry, affecting both productivity and economic sustainability [2]. Moreover, maintaining proper hygiene in dairy cow management is particularly challenging, as mastitis-related production losses and inconsistencies are often exacerbated by the lack of standardized management protocols [3].

Preventing, treating, and eliminating mastitis in dairy herds remains a growing challenge for the livestock industry. Mastitis is primarily caused by bacteria, with more than 150 bacterial species identified as mastitis pathogens. Both Gram-positive and Gram-negative bacteria can be responsible for the disease. Among Gram-positive bacteria, *Staphylococcus* and *Streptococcus* species are the most common, while *Escherichia coli* and *Klebsiella pneumoniae* are the predominant Gram-negative pathogens [4]. These bacteria can colonize and persist in the environment, forming biofilms that further complicate disease management in the dairy industry [5].

To ensure udder hygiene, disinfectants are widely used to sanitize milking equipment and the teats of dairy cows before and after milking, preventing pathogen transmission and reducing biofilm formation. However, the intensive use of these sanitizers has been directly linked to the rise in bacterial resistance, posing a significant obstacle in the control of bovine mastitis [6]. The use of plants and their bioactive compounds for the prevention of bovine mastitis represents a promising alternative in response to the rise in microbial resistance and limitations associated with the prolonged use of conventional chemical disinfectants, such as cytotoxicity. Moreover, there is increasing interest in the development of environmentally sustainable disinfectants and antiseptic products [7,8,9]. When evaluating the efficacy of disinfectants and antiseptics, key parameters such as concentration and contact time must be carefully considered [10]. Comparative studies involving commercial disinfectants have aimed to standardize these variables, testing exposure durations ranging from seconds to hours, in order to optimize udder hygiene protocols in dairy cows [9].

*Punica granatum* L., commonly known as pomegranate, has been extensively studied for its broad-spectrum antimicrobial and anti-inflammatory properties [11]. Numerous studies have demonstrated the beneficial effects of *Punica granatum* in conditions affecting the gastrointestinal, respiratory, visual, and reproductive systems. Additionally, both preclinical and clinical evidence supports the therapeutic potential of pomegranate and its preparations in the management of metabolic disorders, dermatological conditions, wound healing, and oral health [12]. These therapeutic effects are largely attributed to the high content of bioactive polyphenols, particularly ellagitannins such as punicalagin, which have demonstrated potent bactericidal activity against a wide range of pathogenic microorganisms [13,14]. Previous studies have highlighted the potential of *Punica granatum* extracts as natural antiseptic agents, offering an alternative to conventional chemical disinfectants. However, these studies reported bactericidal activity only after a minimum contact time of four hours with the phytoconstituents of *Punica granatum* [15]. Prior work conducted in our laboratory investigated the viability of *Escherichia coli* and *Staphylococcus aureus* suspensions following one hour of exposure to *Punica granatum* extract, using flow cytometry with the Redox Sensor Green Reagent. A significant reduction in fluorescence intensity was observed in bacterial cells treated with the extract compared to untreated controls, suggesting that most of the cells were either non-viable or exhibited impaired metabolic activity [2].

In this study, we evaluated for the first time the short-term bactericidal efficacy of various concentrations of an ethanolic extract of *Punica granatum* in comparison with the most commonly used disinfectants in cattle management. Flow cytometry was employed to assess bacterial membrane integrity; DNA quantification in the supernatant served as a marker of cell lysis; and scanning electron microscopy was used to visualize structural damage to the bacterial membrane. This research aims to provide a deeper mechanistic understanding of *Punica* granatum’s bactericidal potential and explore its viability as a sustainable alternative for infection control in the dairy industry.

## 2. Results

### 2.1. Phytochemical Analysis of the Hydroalcoholic Extract of Punica granatum

**Figure 1:**
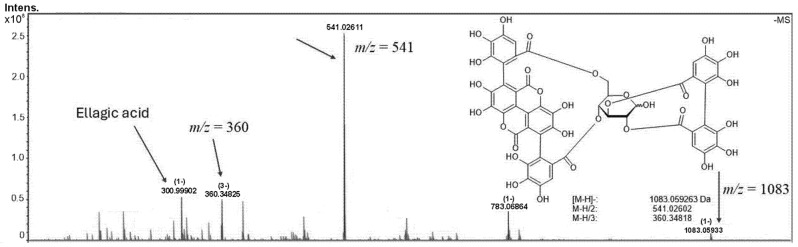
Phytochemical analysis of the hydroalcoholic extract of *Punica granatum* peel. Molecular ions (z 1, 2, and 3) of Punicalagins α and β from (−)-ESI FT-ICR MS (Negative-Ion Electrospray Ionization Fourier Transform Ion Cyclotron Resonance Mass Spectrometry) of *Punica granatum* extracts.

Various methods have been employed for the extraction of phytochemicals from *Punica granatum* peel [16]. In the present study, we selected maceration using a 90% hydroethanolic solvent system, given that ethanol is a safe, non-toxic, and biodegradable solvent. Phytochemical analysis of the hydroalcoholic extract of *Punica granatum* peel revealed the presence of key bioactive compounds, with hydrolyzable tannins, ellagitannins, and punicalagins (α and β) identified as the major constituents. The detection of α and β punicalagins was confirmed by the ions *m*/*z* at 1083.05984 (z = 1), 541.02602 (z = 2), and 360.34818 (z = 3), which confirm the molecular formula C_48_H_26_O_30_ for the molecule (Figure 1). Additionally, ellagic acid (*m*/*z* 300.99902 for C_14_H_6_O_8_), resulting from the hydrolysis of ellagitannins, was also detected, indicating its potential role in the extract’s antimicrobial properties. These findings suggest that the chemical composition of the *Punica granatum* peel extract is rich in polyphenolic compounds, which may contribute to its observed bactericidal activity.

### 2.2. Analysis of the Integrity of Bacterial Cell Membranes After Contact with Punica granatum Extract

The integrity of bacterial cell membranes was assessed using flow cytometry at different time intervals (30, 60, and 120 s) by incorporating propidium iodide (PI), a fluorescent intercalating agent that binds to DNA molecules between their bases. PI cannot passively cross intact cell membranes due to its high molecular weight and relative lipophobicity, which prevents its entry into viable cells with intact membranes. Table 1 and Table 2 summarize the data obtained for *Staphylococcus aureus* and *Escherichia coli* strains, respectively. An increase of over 400% in the median fluorescence intensity was observed in bacterial suspensions treated with various concentrations of the *Punica granatum* extract (50, 25, and 10 mg/mL) at all time points, compared to the median fluorescence intensity of untreated controls. This significant increase in fluorescence intensity suggests a considerable disruption of bacterial membrane integrity, indicating that the extract induces membrane permeabilization and potentially bactericidal effects.

### 2.3. DNA Concentration in Supernatants of Bacterial Cultures After Treatment with P. granatum Extract

**Figure 2:**
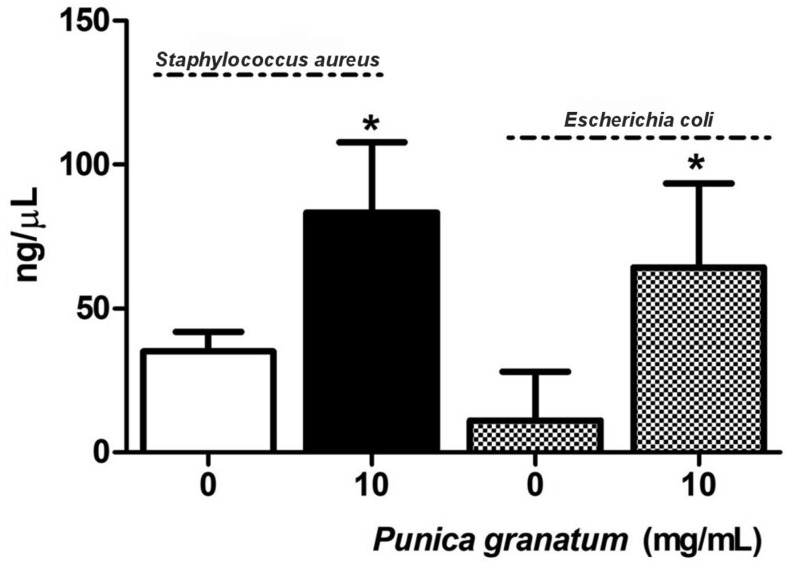
DNA concentration (ng/mL) in bacterial cultures treated with *Punica granatum* extract. Data represent the medians of two experiments performed in triplicate. * *p* < 0.05 when data from samples treated with *Punica granatum* were compared with untreated samples.

The DNA concentration in the supernatants of *Escherichia coli* and *Staphylococcus aureus* cultures was measured following treatment with 10 mg/mL of *Punica granatum* extract for 30 s. This analysis was based on the results obtained from the propidium iodide (PI) impregnation assay by flow cytometry, which indicated a significant disruption of membrane integrity, with an increase of over 200% in fluorescence intensity. As shown in Figure 2, treatment with *Punica granatum* extract resulted in a marked increase in extracellular DNA concentration in the culture supernatants of *Staphylococcus aureus* (83.25 ng/mL) and *Escherichia coli* (64.25 ng/mL), relative to untreated controls (35.20 ng/mL and 11.20 ng/mL, respectively; *p* < 0.05). Although the control value for *Escherichia coli* was near the lower detection range, it remained above the NanoDrop 2000’s reported limit of quantification (LOQ, ~3–5 ng/μL) (Thermo Fisher Scientific, Waltham, MA, USA), ensuring the reliability of the measurement. These findings suggest that exposure to the extract compromises bacterial membrane integrity, facilitating the release of intracellular components, including DNA, into the extracellular environment. The release of nucleic acids is a feature of membrane-disruptive bactericidal activity, aligning with previous reports that link DNA leakage to cell lysis and death.

### 2.4. Ultrastructural Changes of Staphylococcus aureus and Escherichia coli After Treatment with Punica granatum Extract

**Figure 3:**
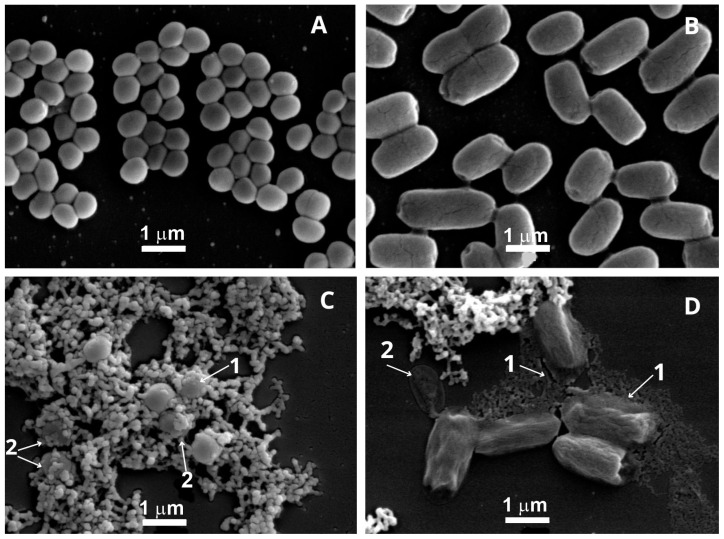
Scanning electron microscopy of *Escherichia coli* and *Staphylococcus aureus* in the absence and presence of *Punica granatum* extract. Suspensions of *Staphylococcus aureus* (**A**) and *Escherichia coli* (**B**) untreated and treated ((**C**,**D**), respectively) with 10 mg/mL of *Punica granatum* extract for 30 s. (1) Rough surface cells. (2) The cells exhibited a wavy outline of the cytoplasmic membrane, suggesting significant shrinkage of the cytoplasm.

**Figure 4:**
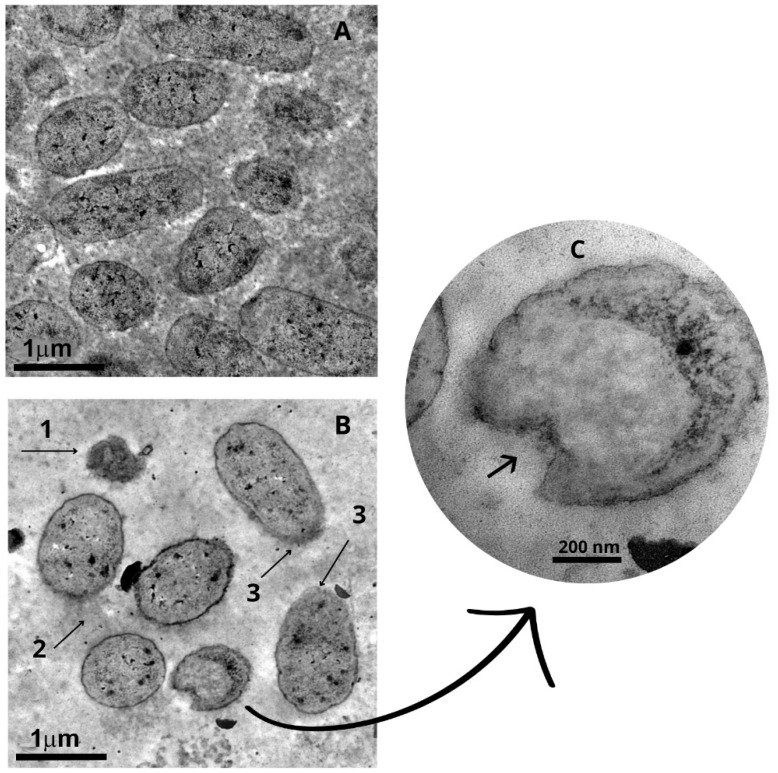
Transmission electron microscopy of *Escherichia coli* in the absence and presence of *Punica granatum* extract. Suspensions of *Escherichia coli* treated with 10 mg/mL of *Punica granatum* extract for 30 s. (**A**) Control (untreated). (**B**) (1) Dead cells, (2) leakage of intracellular contents, (3) change in membrane integrity. (**C**) Magnification 80,000× with a 200 nm calibration bar, showing changes in the cell wall and release of cytoplasmic contents.

**Figure 5:**
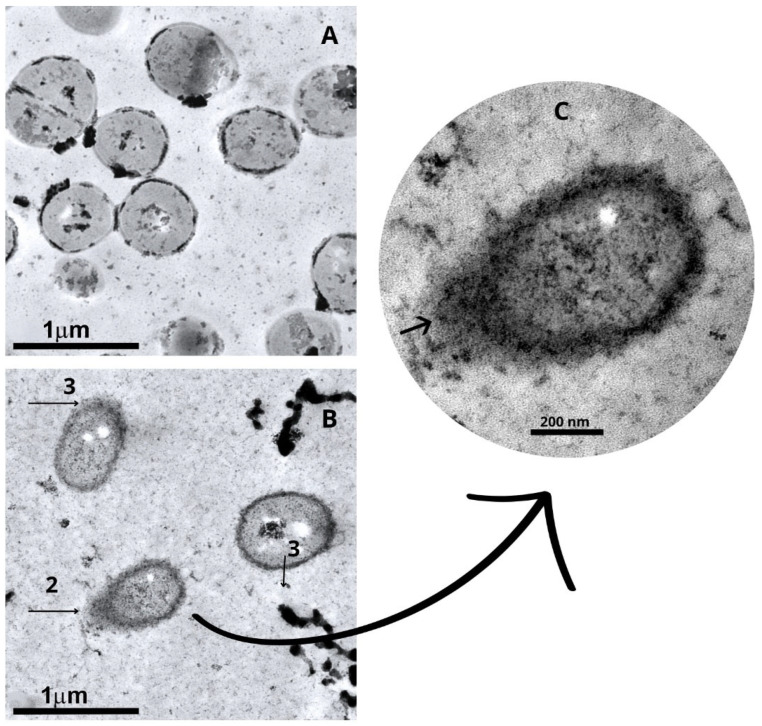
Transmission electron microscopy of *Staphylococcus aureus* in the absence and presence of *Punica granatum* extract. Suspensions of *Staphylococcus aureus* treated with 10 mg/mL of *Punica granatum* extract for 30 s. (**A**) Control (untreated). (**B**) (2) Leakage of intracellular contents, (3) change in membrane integrity. (**C**) Magnification 80,000× with a 200 nm calibration bar, showing changes in the cell wall and release of cytoplasmic contents.

Ultrastructural alterations induced by *Punica granatum* extract were investigated using scanning (SEM) and transmission (TEM) electron microscopy. A total of 15 representative fields per condition were analyzed. SEM examination revealed marked surface deformation in both *Staphylococcus aureus* and *Escherichia coli* cells following treatment with 10 mg/mL of the extract for 30 s. Untreated *Staphylococcus aureus* cells exhibited their characteristic spherical morphology, with smooth and uniform surfaces (mean diameter: 0.66 µm; estimated area: 0.34 µm^2^), whereas treated cells appeared enlarged (mean diameter: 0.88 µm; area: 0.61 µm^2^), less uniform in shape, and displayed irregular, rough surfaces, corresponding to a ~78% increase in cell area. Similarly, *Escherichia coli* cells, which typically display a rod-like shape, showed a notable expansion in length following extract exposure (from 1.55 µm to 2.22 µm), with a corresponding increase in surface area from 1.36 µm^2^ to 1.95 µm^2^ (~43% increase), assuming a fixed height of 0.88 µm. In addition to size alterations, treated cells displayed substantial membrane distortion, including wavy outlines and compromised integrity suggestive of cytoplasmic shrinkage and lysis. TEM further corroborated these findings, revealing extensive membrane rupture, cytoplasmic leakage, and a significant reduction in viable cell counts per field, alongside the frequent presence of lysed or ghost cells. Quantitative image analysis performed using the EBImage package—V. 4.32.0 (R) confirmed an ~80% reduction in intact bacterial cells per field following treatment. These data collectively support that the *Punica granatum* extract rapidly compromises bacterial cell structure and integrity, strongly contributing to its bactericidal mechanism of action (Figure 3, Figure 4 and Figure 5).

### 2.5. Comparison of Bacterial Survival After Treatment with Different Concentrations of Extract and Disinfectants

The bacterial survival data presented in Table 3 reflect the outcomes of treatments with varying concentrations of *Punica granatum* extract (50 mg/mL, 25 mg/mL, and 10 mg/mL), as well as conventional disinfectants, chlorhexidine (2.0% and 0.5%) and sodium hypochlorite (NaOCl, 2.5% and 0.5%), applied for 30 s and evaluated via broth microdilution. Our results demonstrate that *Punica granatum* extract at 50 mg/mL and 25 mg/mL exerted bactericidal effects against *Escherichia coli* and *Staphylococcus aureus* within this brief contact time. In contrast, the 10 mg/mL concentration showed no bactericidal activity against *Escherichia coli*, and only a 79.4% reduction in *Staphylococcus aureus* viability, suggesting a concentration-dependent efficacy. Chlorhexidine was effective at both tested concentrations, corroborating its known potent and broad-spectrum antimicrobial activity. Sodium hypochlorite, however, was less effective at 0.5%, failing to achieve a bactericidal effect against *Staphylococcus aureus* and reducing *Escherichia coli* viability by only 88.9%, highlighting the limitations of lower concentrations and organic matter interference.

While these results establish a comparable or even superior short-contact bactericidal performance of *Punica granatum* extract relative to standard disinfectants, important contextual factors require consideration. The modes of action differ significantly: phenolic-rich plant extracts primarily disrupt membrane integrity and interfere with intracellular targets, while chlorhexidine disrupts osmotic balance and enzyme function, and NaOCl acts via oxidative protein damage. Additionally, the cytotoxicity profiles and environmental persistence of these agents diverge. For instance, chlorhexidine’s efficacy is pH-dependent and reduced in the presence of organic matter, and NaOCl poses environmental and tissue toxicity concerns.

The concentrations of 0.5% and 2.0% for chlorhexidine, and 2.5% for NaOCl, were selected based on their frequent use in pre- and post-dipping protocols in dairy cattle, as documented in prior field studies [9,17]. Nonetheless, future work should include a clearer justification of concentration choices, standardized across studies, and incorporate cytotoxicity assays and cost-effectiveness comparisons. A cost–benefit analysis encompassing production, safety, environmental impact, and antimicrobial resistance risk will be essential for translating these findings into practical, sustainable applications in livestock hygiene management.

## 3. Discussion

The use of disinfectants to clean the teats of dairy cows before and after milking is an essential sanitary practice that significantly reduces the local microbial load, thereby minimizing the transmission of mastitis-causing agents. This management measure has been proven to effectively eliminate microorganisms, particularly bacteria, from the teat surface [18]. The effectiveness of disinfectants is influenced by several factors, including concentration, exposure time, temperature, presence of organic matter, the specific area being disinfected, the sensitivity of microorganisms, and the training of cow handlers.

Commercially available chemical disinfectants are commonly employed during pre- and post-milking hygiene procedures. Since no single disinfectant can be deemed ideal for all circumstances, the selection process considers the composition, cost, and effectiveness of the disinfectant, alongside its safety for both animals and humans. Among the most widely used disinfectants are chlorhexidine, iodine, sulfonic acid, chlorine, sodium hypochlorite, and chlorous acid. To mitigate potential adverse effects, emollients such as glycerin, lanolin, propylene glycol, sorbitol, vegetable and mineral oils, and collagen are often added to these disinfectants [9,10,11,12,13,14,15,16,17].

Antimicrobial resistance (AMR) is a significant global health issue, posing a serious threat to human health, food security, and safety, as emphasized by the World Health Organization (WHO). In response to this challenge, natural sanitizers have emerged as promising alternatives for the treatment of bovine mastitis. These include essential oils, vegetable oils, plant extracts, bacteriocins, and phytoderivatives. These substances are particularly effective due to the complexity of their molecular structures, which make it difficult for bacteria to develop resistance. As such, they offer a viable and efficient solution to combat antimicrobial resistance in veterinary and agricultural settings [8,12].

*Punica granatum* (pomegranate) is a plant native to Asia, specifically from the region of Iran to the Himalayas in northwest Asia, and is now cultivated worldwide, including in Brazil [19]. This species holds significant pharmacological and phytotherapeutic value, with various parts of the plant, such as flowers, leaves, stem and root bark, seeds, young stems, fruits, and their extracts and juice, being used in traditional therapies [20]. The phenolic substances in *Punica granatum* are primarily derived from the shikimic acid and phenylpropanoid pathways. These compounds feature an aromatic ring with one or more hydroxyl groups and are mainly classified into flavonoids, phenolic acids, and tannins. The most abundant tannin in *Punica granatum* fruit is punicalagin, with its concentration varying depending on the extraction process [19,21]. Other notable tannins found in *Punica granatum* include gallic acid, ellagic acid, chlorogenic acid, and punicalin [22].

Pomegranate products, including juice and extracts, generally show good stability when stored at refrigerated temperatures (around 4 °C). Studies have demonstrated that the total phenolic content and antioxidant capacity remain relatively stable or may even increase slightly during refrigerated storage for up to about 60 to 90 days. Pomegranate powder, when stored in airtight containers at 4 °C, retains its flavor, color, and nutritional quality for up to 6 months [23,24]. Although numerous studies have investigated the bactericidal properties of pomegranate peel extract, its bactericidal potential over short periods and its possible use as a disinfectant remain underexplored. Phytochemical analysis of the extract from *Punica granatum* peel supports previous findings from our research group [2], which identified the major antimicrobial compounds in the extract, including punicalagins, ellagic acid, and gallic acid. The present study provides novel evidence that a short 30 s exposure to an ethanolic extract of *Punica granatum* peel exerts a measurable bactericidal effect against *Staphylococcus aureus* and *Escherichia coli*, two of the principal etiological agents of bovine mastitis. This finding is particularly relevant given the increasing demand for effective, sustainable alternatives to conventional disinfectants used in dairy herd management.

The bactericidal action observed is consistent with the phytochemical profile of the extract, which is rich in punicalagins, ellagic acid, and gallic acid, compounds previously associated with membrane-disruptive and redox-modulating properties. Flow cytometry analyses revealed significant alterations in bacterial membrane permeability, corroborated by increased extracellular DNA levels and ultrastructural damage observed by scanning electron microscopy. These complementary approaches confirm that the extract exerts its bactericidal effects, at least in part, via disruption of the bacterial envelope.

Although previous studies have demonstrated the bactericidal activity of punicalagin, these effects typically required prolonged exposure times (≥4 h) [8]. In contrast, our results show that robust antimicrobial effects can be achieved within substantially shorter contact durations when using a crude hydroalcoholic extract of *Punica granatum*. This suggests a possible synergistic interaction among the extract’s multiple phytochemical constituents. While these findings align with the existing literature, they underscore the need for further investigation into the specific contributions of the individual purified compounds. Notably, the cellular disruptions observed, affecting membrane integrity and viability, were stimulated by the whole extract, emphasizing its practical relevance. Its efficacy under short exposure times enhances its suitability for on-farm applications, where rapid action is critical. Moreover, as a biodegradable and non-toxic alternative to widely used disinfectants such as sodium hypochlorite and chlorhexidine, *P. granatum* extract offers a promising solution to challenges associated with environmental persistence, cytotoxicity, and the rise in antimicrobial resistance.

The SEM and TEM images revealed ultrastructural changes in the bacterial cells, including a reduction in size, disrupted membranes, irregular cell walls, leakage of cytoplasmic content, fragmentation of cells, and a decrease in the number of cells per field when compared to untreated cultures. Protein aggregates were observed, likely resulting from the leakage of intracellular content and proteins present in the culture medium. Studies have shown that polyphenols can interact with proteins through hydrophobic interactions and hydrogen bonds, forming complexes [25,26]. The most widely accepted mechanisms by which plant-derived phenolic compounds exert antimicrobial activity involve interactions at the level of the bacterial membrane. Numerous studies have reported dose-dependent perturbations to microbial membranes, ranging from transient increases in permeability to complete structural disruption, ultimately leading to the leakage of intracellular contents [27]. In addition to these membrane-level effects, other mechanisms have been proposed, including the dissipation of membrane potential, inhibition of key metabolic enzymes, cytoplasmic acidification, coagulation of intracellular components, and biofilm inhibition [28,29,30].

While the results of the present study point to membrane rupture as a major bactericidal mechanism of *Punica granatum* phenolic compounds, evidenced by increased membrane permeability, elevated extracellular DNA, and morphological damage, further investigations are needed to resolve whether these effects stem from direct interactions with membrane lipids and proteins or from indirect destabilization via intracellular targets. These may include the disruption of membrane potential, pH homeostasis, or enzymatic activity. Major compounds like punicalagin contributed to the inactivation of bacterial extracellular proteins by interacting with sulfhydryl groups and reducing pH, generating protons, and precipitating proteins from the protoplast and plasma membrane. This process alters their functionality, leading to the loss of cytoplasmic content. Additionally, ellagic and gallic acids, isomers of punicalagin, play a role in modulating plasma membrane potential. By acidifying the interface, they disrupt membrane protein pumps and channels, inhibiting nutrient transport and enzymatic activity, which results in bacterial growth inhibition, reduced resistance to antimicrobial agents, and eventual cell death [14,31].

These observations prompted us to evaluate the efficacy of different concentrations of *Punica granatum* extract in comparison with the most commonly employed disinfectants for pre- and post-dipping procedures, namely, sodium hypochlorite and chlorhexidine. Chlorhexidine is a widely used broad-spectrum biocide characterized by low cytotoxicity and rapid bactericidal action, with effective exposure times ranging from 30 s to 2 min. Its mechanism of action involves increased cytoplasmic membrane permeability, the precipitation of intracellular proteins, disruption of osmotic balance, interference with energy metabolism and cell division, and inhibition of membrane-bound ATPases and anaerobic respiration [32]. However, despite its clinical advantages, chlorhexidine exhibits reduced efficacy in the presence of organic matter and is pH-dependent, which can limit its effectiveness under field conditions [33]. Sodium hypochlorite, in turn, is a potent oxidizing agent known for its broad antimicrobial activity. It exerts its effect primarily through the oxidative denaturation of cellular proteins and membrane lipids, ultimately leading to membrane rupture and cell death [10]. Despite their effectiveness, both disinfectants raise concerns regarding environmental persistence and potential cytotoxicity, reinforcing the need for alternative, biodegradable, and non-toxic solutions such as plant-derived antimicrobials. In post-dipping asepsis, which aims to maintain an antiseptic effect while preserving the well-being of newly milked cows, the teat canal remains open for some time after milking. High concentrations of disinfectants and prolonged exposure could irritate or even cause inflammation in the teats [9,34].

Previous in vivo studies have demonstrated that oral administration of *Punica granatum* ethanolic extracts at doses up to 2000 mg/kg in female rats did not induce observable toxic effects, indicating a favorable safety profile under those conditions [25]. Moreover, the consumption of pomegranate peel infusions by both humans and animals further supports its potential as a safe bioactive agent in general use [7,35]. However, these findings pertain to systemic exposure via ingestion and do not directly address the safety of topical or mucosal application, which is the intended mode of use in dairy cattle udder hygiene. Therefore, any assumption of safety for external application, particularly on bovine teats, remains premature.

While the bioactive metabolites present in *Punica granatum* extract have well-documented bactericidal, anti-inflammatory, and tissue-regenerative properties, the cytotoxic and irritant potential of the extract when applied dermally or on mucosal surfaces has not been assessed in this study. This is a critical gap, as the anatomical and physiological characteristics of bovine teats may pose unique sensitivities to topical agents. Furthermore, although the polar nature of *Punica granatum* phytochemicals facilitates their solubility, dilution, and incorporation into aqueous formulations—supporting potential scalability for industrial application—comprehensive toxicological evaluations remain essential. Specifically, future studies must assess the extract’s cytotoxicity at the concentrations and exposure durations used here, as well as conducting in vivo evaluations of dermal irritation, percutaneous absorption, and local tolerability. These steps are crucial to ensure biocompatibility and safety prior to the development of formulations intended for field use in veterinary and agricultural settings.

## 4. Materials and Methods

### 4.1. Plant Material and Extract Preparation

Pomegranate (*Punica granatum* L., Lythraceae) samples were collected from Sítio Boa Vista in Taquaritinga, São Paulo, Brazil (21°24′6″ S, 48°27′8″ W). The species was taxonomically identified based on morphological characteristics of branches, flowers, and fruits, and a voucher specimen (HUNI 6241) is deposited in the Herbarium Prof. Jorge Pedro Pereira Carauta at the Federal University of the State of Rio de Janeiro (UNIRIO). Following collection, the fruits were thoroughly washed with detergent and air-dried. The peels were separated from the pulp and seeds, then stored at −20 °C until further processing. Prior to extraction, the peels were freeze-dried for 24 h and ground into a fine powder using a paddle mill (IKA A11 Basic, IKA, Staufen, Germany). For hydroalcoholic extraction, 100 g of pomegranate peel powder was macerated in 1000 mL of 90% (*v*/*v*) ethanol for 10 days, with daily agitation, in a light-protected and dry environment. The mixture was subsequently filtered using a Büchner funnel, and the ethanol was evaporated under reduced pressure at 45 °C using a rotary evaporator. The resulting dried extract was stored in sterilized, airtight containers at 4 °C until use. The extraction process yielded 252 g of dry extract from 500 g of freeze-dried pomegranate peels.

### 4.2. Phytochemical Analysis of Punica granatum Extract

The phytochemical composition of the ethanolic peel extract of *Punica granatum* was analyzed using Fourier Transform Ion Cyclotron Resonance Mass Spectrometry (FT-ICR MS) (Fourier, Shanghai, China) with an electrospray ionization (ESI) source in negative mode. The analysis was conducted on a Solarix XR 7 T mass spectrometer (Bruker, Billerica, MA, USA). For the analysis, the extract was solubilized in a 1:1 (*v*/*v*) water/acetonitrile solution and diluted to a final concentration of 1 mg/mL in the same solvent mixture, which also contained 0.1% ammonium hydroxide (NH_4_OH) to facilitate ionization. The mass spectrum was acquired in a mass-to-charge ratio (*m*/*z*) range of 75–1200 *m*/*z*. The ESI source was operated under the following conditions: nebulizer gas pressure at 1.0 bar, capillary voltage set between 3.0 and 3.5 kV, and a capillary transfer temperature of 25 °C. Data acquisition and subsequent spectral analysis were performed using the Compass data analysis software package version 5.0 (Bruker, Billerica, MA, USA).

### 4.3. Bacterial Strains

The bacterial strains utilized for screening in this study were *Escherichia coli* (INCQS 00219, ATCC 8739) and *Staphylococcus aureus* subsp. *aureus* (INCQS 00039, ATCC 6538), both of which are commonly associated with clinical and environmental cases of bovine mastitis. These strains were selected due to their relevance in mastitis pathogenesis and their widespread occurrence in both clinical and environmental settings, making them ideal candidates for evaluating the bactericidal efficacy of the *Punica granatum* extract.

### 4.4. Analysis by Flow Cytometry of Membrane Integrity in Staphylococcus aureus and Escherichia coli Strains After Treatment with Punica granatum Extract

To assess the impact of the *Punica granatum* extract on bacterial membrane integrity, flow cytometry was employed using propidium iodide (PI), a DNA intercalating agent that serves as a marker for alterations in membrane permeability. Bacterial suspensions, each with a density of 10^2^ CFU/mL, were treated with varying concentrations of *Punica granatum* extract (50, 25, and 10 mg/mL) for 30 s. After treatment, the bacterial suspensions were analyzed by flow cytometry using a BD FACScalibur™ Flow Cytometer (BD Biosciences, Franklin Lakes, NJ, USA), where 20,000 cells were assessed per sample. Data acquisition and analysis were performed using CellQuest software (version 6.0). The proportion of PI-positive cells, indicative of compromised membrane integrity, was used to determine the bactericidal effects of the extract and to compare its efficacy across the different concentrations tested. The assays to evaluate the integrity of the membranes were performed by flow cytometry using propidium iodide, a DNA intercalant, which is a marker of alteration in membrane permeability. Bacterial suspensions with a density of 10^2^ CFU/mL were treated with different concentrations of *Punica granatum* extract (50, 25, and 10 mg/mL) for 30 s. Afterwards, the bacterial suspensions were subjected to flow cytometry where 20,000 cells were analyzed by the BD FACScalibur™ Flow Cytometer. Data analyses were performed using CellQuest software version 6.0.

### 4.5. Quantification of DNA in the Supernatant of Bacterial Strain Cultures After Treatment with Punica granatum Extract

To evaluate the extent of bacterial membrane damage and DNA release, bacterial suspensions of *Escherichia coli* and *Staphylococcus aureus* were prepared at a concentration of 10^2^ CFU/mL and treated with varying concentrations of *Punica granatum* extract (50, 25, and 10 mg/mL) for 30 s. Control samples were left untreated and incubated for 30 s at room temperature under identical conditions. Following treatment, the bacterial suspensions were centrifuged at 4500 rpm for 10 min at 4 °C, and the resulting supernatants were carefully collected for DNA analysis. DNA was extracted from the supernatants using the FastDNA Spin Kit for Soil (MP Biomedicals, Santa Ana, CA, USA), following the manufacturer’s protocol. DNA concentration was quantified using a NanoDrop 2000 spectrophotometer (Thermo Fisher Scientific, Waltham, MA, USA), which allowed for precise measurement of DNA released into the supernatant [36] as a result of membrane disruption induced by the *Punica granatum* extract.

### 4.6. Evaluation of Morphological and Ultrastructural Characteristics of Bacterial Strains After Treatment with Punica granatum Extract

Bacterial suspensions of approximately 10⁸ CFU/mL were prepared and treated with 10 mg/mL of *Punica granatum* extract derived from fruit peels. Untreated controls were also included in the study for comparison. Following a 30 s exposure period, the bacterial suspensions were centrifuged at 4500 rpm for 10 min at 4 °C. The supernatants were discarded, and the bacterial pellets were resuspended in 500 µL of Karnovsky buffer (4% paraformaldehyde, 2.5% glutaraldehyde) for fixation. Subsequent ultrastructural analysis was conducted using both scanning electron microscopy (SEM) and transmission electron microscopy (TEM), following the protocols established [26,27]. For SEM, the samples were metallized and analyzed with a JEOL-JSM-6390-LV electron microscope (JEOL, Akishima, Tokyo, Japan). For TEM, the samples were examined using a JEM-1200EX electron microscope (JEOL, São Paulo, Brazil). EBImage, an R package designed for image processing and analysis, was used to read, write, process, and analyze images, including the segmentation of cells and the extraction of quantitative cellular descriptors [37].

### 4.7. Determination of Bacterial Survival After Treatment with Different Concentrations of Extract and Disinfectants

Sodium hypochlorite and chlorhexidine were included in the study as reference disinfectants to enable a comparative evaluation of the efficacy of the *Punica granatum* extract. The selection of these agents, as well as their respective concentrations, was based on established protocols commonly employed in dairy herd management. These protocols are supported by the literature, which identifies these disinfectants as among the most widely used in pre- and post-milking hygiene practices due to their broad-spectrum bactericidal activity and rapid onset of action under practical farm conditions [9,17]. To assess bacterial survival, bacterial suspensions of *Escherichia coli* and *Staphylococcus aureus*, each with a density of approximately 10^2^ CFU/mL, were treated for 30 s with varying concentrations of *Punica granatum* extract (50 mg/mL, 25 mg/mL, and 10 mg/mL), sodium hypochlorite (2.5% and 0.5%), and chlorhexidine (2.0% and 0.5%). Following treatment, the suspensions were diluted in a new 96-well microplate. Specifically, 180 µL of sterile phosphate-buffered saline (PBS) and 20 µL of the bacterial cultures with the different concentrations of the extract and disinfectants were added to each well, followed by serial dilutions from 10^−1^ to 10^−9^. To determine the colony-forming units per milliliter (CFU/mL), aliquots of 10 µL from each dilution were plated in triplicate onto Müeller–Hinton agar medium in a spot-seeding pattern. The plates were incubated at 35 ± 2 °C for 24 h, after which the colonies were counted to quantify bacterial survival. The results were expressed in CFU/mL, and all experiments were performed in triplicate to ensure the reliability of the data.

### 4.8. Statistical Analysis

The results were presented as the median with the 1st and 3rd quartiles (1st Q/3rd Q) of the obtained values. Statistical analyses were conducted using GraphPad Prism 5 software. Differences between groups were assessed using the Dunn’s Multiple Comparison Test, which was applied following a non-parametric test to account for the data distribution. Statistical significance was considered at a *p*-value of <0.05.

## 5. Conclusions

*Punica granatum* extract exhibits bactericidal effects primarily through the disruption of bacterial cell membranes. These effects are largely attributed to its major phenolic constituents, punicalagin, ellagic acid, and gallic acid, which act synergistically to exert bactericidal activity. In addition to its bactericidal potential, *Punica granatum* peel is known for its anti-inflammatory, tissue regenerative, and even anti-tumor properties, further underscoring its therapeutic value. The present study highlights the extract’s promise as a natural, biodegradable alternative to conventional teat disinfectants. In particular, the demonstrated rapid bactericidal action within 30 s reveals *Punica granatum* extract as a strong candidate for inclusion in pre- and post-milking teat-dip solutions aimed at the prevention and control of bovine mastitis. However, before such formulations can be implemented in field conditions, further studies are required to evaluate their cytotoxicity at the concentrations used, as well as in vivo assessments of dermal irritation, skin absorption, and long-term biocompatibility. These investigations will be critical to ensuring safety and efficacy, facilitating the translation of this natural extract into practical, farm-ready applications.

## Figures and Tables

**Table 1:** Effect of *Punica granatum* extract on the integrity of *Escherichia coli* cell membranes *.

Time (s)	Control	50 mg/mL	25 mg/mL	10 mg/mL
30	13.1 (12.4/20.9)	214.8 (167.0/235.0)	201.7 (118.8/239.3)	91.4 (87.8/179.4)
60	18.6 (13.3/22.3)	197.7 (126.4/269.0)	189.4 (86.0/239.0)	111.4 (61.0/118.6)
120	18.5 (13.0/22.5)	218.7 (100.9/250.3)	181.1 (67.9/245.8)	118.6 (82.0/129.8)

* Data represent the median (1st quartile/3rd quartile) of three experiments where 20,000 cells were analyzed by flow cytometry.

**Table 2:** Effect of *Punica granatum* extract on the integrity of *Staphylococcus aureus* cell membranes *.

Time (s)	Control	50 mg/mL	25 mg/mL	10 mg/mL
30	12.6 (11.6/24.9)	179.4 (164.0/198.1)	138.9 (129.8/283.9)	129.8 (63.8/189.4)
60	12.5 (12.1/14.5)	191.1 (120.8/212.9)	115.5 (79.8/177.4)	121.9 (107.5/220.7)
120	13.5 (11.6/41.4)	196.3 (148.6/199.9)	64.36 (50.5/160.9)	82.79 (77.0/259.5)

* Data represent the median (1st quartile/3rd quartile) of three experiments where 20,000 cells were analyzed by flow cytometry.

**Table 3:** Antimicrobial efficacy of *Punica granatum* extract and commercial disinfectants against *Staphylococcus aureus* and *Escherichia coli*. Determination of colony-forming units per milliliter (CFU/mL) of *S. aureus* and *Escherichia coli* suspensions following treatment with various concentrations of *Punica granatum* extract and commercial disinfectants *.

Bacterial Species	Control	*Punica granatum* Extract (mg/mL)			Chlorhexidine (%)		Sodium Hypochlorite (%)	
	0	50	25	10	2	0.5	2.5	0.5
*Staphylococcus aureus*	1.0 × 10^2^ (1.0/1.7)	0.0	0.0	0.0	0.0	0.0	0.0	1.3 × 10^2^ (1.0/1.6)
*Escherichia coli*	6.3 × 10^2^ (1.0/1.7)	0.0	0.0	1.3 × 10^2^ (0/1.3)	0.0	0.0	0.0	0.7 × 10^2^ (0.3/1.3)

* The data represent the median (1st quartile/3rd quartile) from three independent experiments, each conducted in triplicate.

## Data Availability

The original contributions presented in this study are included in the article. Further inquiries can be directed to the corresponding author.

## Abbreviations

The following abbreviations are used in this manuscript:

SEM	Scanning electron microscopy
TEM	Transmission electron microscopy
AMR	Antimicrobial resistance
WHO	World Health Organization
PI	Propidium iodide
